# Application of two-component neural network for exchange-correlation functional interpolation

**DOI:** 10.1038/s41598-022-18083-1

**Published:** 2022-08-19

**Authors:** Alexander Ryabov, Iskander Akhatov, Petr Zhilyaev

**Affiliations:** 1 Center for Materials Technologies, Skolkovo Institute of Science and Technology, Bolshoy Boulevard 30, bld. 1, Moscow, 121205 Russia; 2 Moscow Institute of Physics and Technology (State University), Institutskiy per. 9, Dolgoprudny, Moscow Region, 141700 Russia

**Keywords:** Density functional theory, Computational science

## Abstract

Density functional theory (DFT) is one of the primary approaches to solving the many-body Schrodinger equation. The essential part of the DFT theory is the exchange-correlation (XC) functional, which can not be obtained in analytical form. Accordingly, the accuracy improvement of the DFT is mainly based on the development of XC functional approximations. Commonly, they are built upon analytic solutions in low- and high-density limits and result from quantum Monte Carlo or post-Hartree-Fock numerical calculations. However, there is no universal functional form to incorporate these data into XC functional. Instead, various parameterizations use heuristic rules to build a specific XC functional. The neural network (NN) approach to interpolate the data from higher precision theories can give a unified path to parametrize an XC functional. Moreover, data from many existing quantum chemical databases could provide the XC functional with improved accuracy. We develop NN XC functional, which gives exchange potential and energy density without direct derivatives of exchange-correlation energy density. Proposed NN architecture consists of two parts NN-E and NN-V, which could be trained in separate ways, adding new flexibility to XC functional. We also show that the developed NN XC functional converges in the self-consistent cycle and gives reasonable energies when applied to atoms, molecules, and crystals.

## Introduction

Since its emergence, density functional theory (DFT)^1,2^ serves as one of the primary methods of solving the many-body Schrodinger equation. The main theoretical bottleneck in DFT theory is the unknown form of the exchange-correlation (XC) functional. Therefore, the progress in developing more accurate XC functionals reveals more possibilities for using DFT in cases where high accuracy of quantum-mechanical calculations is required. Information for constructing XC functionals is taken from numerical calculations using quantum Monte Carlo or post-Hartree-Fock^3,4^. The influential Monte Carlo (MC) simulations of the uniform electron gas (UEG) by Ceperley and Adler^3^ led to the creation a number of practical local density approximations (LDAs)^5–7^. The next big success in reaching better accuracy was achieved by the generalized gradient approximation (GGA), which takes into consideration local gradients of electron density^8–10^. This improvement sufficiently increased the capability of DFT to characterize systems with inhomogeneous electron densities. Calculations made by post-Hartree-Fock methods were also used to improve the quality of the XC functionals for molecular systems^4,11^. The search for advanced XC functional is ongoing, and, still a very active direction of research^12–15^.

Regardless of the evident triumph of the LDA and GGA, their development is a highly complex procedure that involves many heuristics stages. The XC functional’s form is generally specified by physical insights (the local nature of the interaction, perturbation approach, analytical solutions in limiting cases, etc.), and a set of adjustable parameters^7,10^. Such inflexible functional form makes it difficult to include more numerical results from modern quantum MC^16,17^ and post-Hartree-Fock calculations^18^, which could lead to increased XC functional accuracy. Therefore it may be fruitful to use an adaptive XC functional form that, on the one hand, facilitates the incorporation of numerical calculations and, on the other hand, enable to include the physical insights into it.

**Figure 1:**
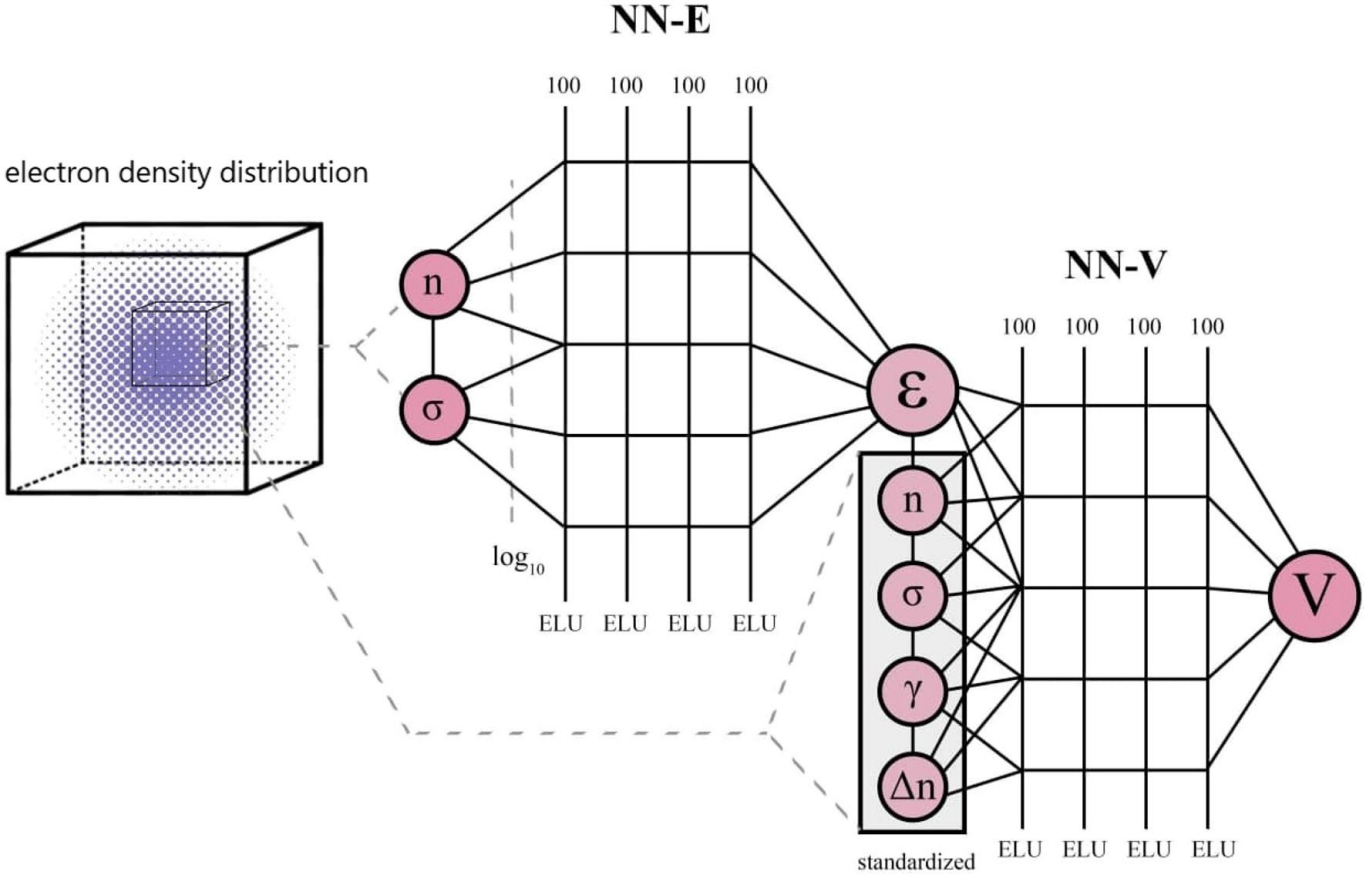
Topology of the XC neural network. It consists of two parts: NN-E predicts $\varepsilon _{xc}$ and NN-V predicts $v_{xc}$. Each part of the neural network consists of 4 layers each of 100 neurons. For both parts information on local density and its derivatives is needed.

One perspective candidate for the flexible XC functional form is the neural network (NN), which provides a universal approach to approximate any functional relationship^19^. Analytical information could be also included in the NN as a synthetically generated part of the dataset. NN was first utilized as a functional form for XC potential by Tozer et al.^20^. After that, several studies have been addressed the possibility of using NN to approximate XC functionals form^21–26^. Work by Nagai et al.^22^ is especially worthy of note. They presented the first working NN XC functional, which gives better accuracy than traditional ones.

Despite significant advances in the development of XC functionals based on NNs, a wide range of issues remain. Namely, how to relate the exchange-correlation energy density ($\varepsilon _{xc}$) and the exchange-correlation potential (XC potential, $V_{xc}$)? In the analytical description, such connection is provided by a standard differentiation, which is sometimes tedious but straightforward. But when new features are included in the NN, for example, the logarithm of density (standard feature scaling procedure) or local Hartree-Fock exchange energy densities^27^, it is not clear how to incorporate them into the connection between XC potential and corresponding energy density. It is also essential to find a way to include physical insight into XC functionals, such as asymptotic analytical solutions and conservation laws. Another issue is an optimal NN architecture and feature selection for presenting XC functional. So far, no detailed comparison has been made of various NN architectures on the same training data.

In this study we focus on constructing NN XC functional that output both $\varepsilon _{xc}$ and $V_{xc}$. The developed NN consists of two parts: one part is used to evaluate $\varepsilon _{xc}$ (NN-E), and another approximates $V_{xc}$ (NN-V). They connected in such a way that the output of NN-E is one of the input features for NN-V (see Fig. 1). Proposed architecture is also “economical”, since one back-propagation step does not need to traverse the entire mesh, and it is calculated only at one point. In other practical approaches^22,27^, for one step of the neural network, it is necessary to calculate the total exchange-correlation energy of the system, which significantly increases memory consumption and therefore leads to limitations for training and evaluation of neural networks with a large number of parameters. Direct differentiation of features to obtain the exchange-correlation functional from the density of the exchange-correlation energy can be quite a complex and confusing problem. In particular, if the features are semi-local or global, obtaining the final analytic expression is non-trivial. An example is the exchange energy density as a feature in the work^27^. Local coordinate transformations only complicate the task.

The training dataset was obtained from the DFT calculation of crystalline silicon, benzene, and ammonia with PBE XC functional^10,28^. After XC NN training, we implemented it into Octopus DFT code^29–31^ and conducted self-consistent cycle calculations of train/test systems and atoms and molecules from IP13/03 dataset^32^. The mean relative error of total and XC energies on training samples is on the order of $0.001\%$. The same errors on crystals and molecules that were not used to train XC NN increased by the order magnitude but were still small, around $0.01\%$. Such a small relative error indicates that the proposed architecture could be successfully used for XC functional form representation. The key feature of the proposed NN architecture is that the weights of NN-V are pre-trained on $\varepsilon _{xc} \rightarrow V_{xc}$ mapping and fixed during learning on new training data. It allows being sure that the output of NN-E is indeed the $\varepsilon _{xc}$, because the relation between $\varepsilon _{xc}$ and $V_{xc}$ is preserved.

It is also should be noted that the boundary conditions are included by using extra datasets. They are synthetically generated to fulfill given boundary conditions. In present case it was $\varepsilon _{xc} \rightarrow 0$ given that electron density (*n*) is vanishing, and $\varepsilon _{xc} \rightarrow \varepsilon _{xc}^{LDA}$ given that gradient modulus squared of electron density ($\sigma$) is also approaching zero.

**Figure 2:**
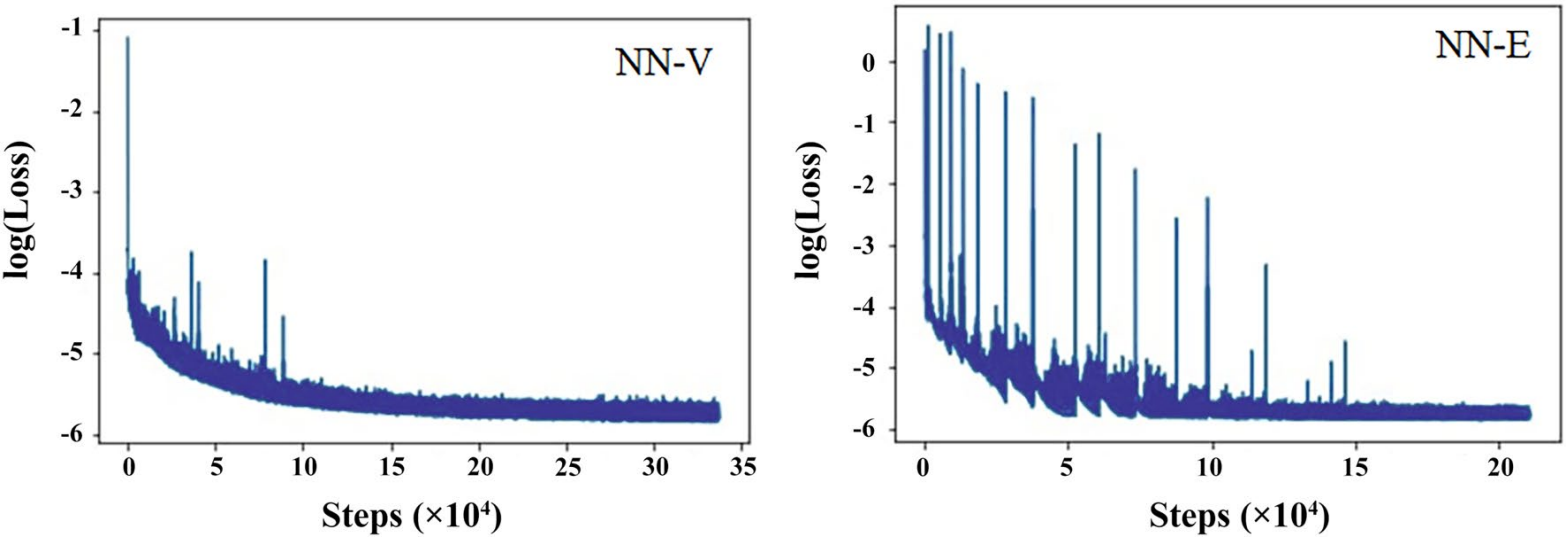
Training curves of XC neural network. The left one corresponds to NN-V training, where log(Loss) is the logarithm of loss (2). The right one corresponds to NN-E training where log(Loss) is the logarithm of loss with boundary conditions (3). Outliers on the training curves are related with adjustable learning rate used for optimization algorithm.

This paper is outlined as follows: In “Methods” section, we describe the data generation process, NN topology, features and hyperparameters used. In the “Results” section, we first show the results of NN training, features distributions, and evaluated metrics; in the following, we demonstrate NN XC’s application in DFT calculations on atoms and molecules that were not presented in the training dataset. In “Conclusion” section, we summarize our results and suggest following research steps that could be done with the developed NN XC pipeline, with a particular emphasis on integrating data from high-level methods such as quantum MC and post-HF calculation.

## Methods

The proposed neural network architecture consists of two parts: the NN-E and the NN-V. The NN-E serves to obtain the exchange-correlation energy density ($\varepsilon _{xc}$), and the NN-V allows calculating of the exchange-correlation potential ($V_{xc}$) from the corresponding energy using the output of NN-E as one of the neurons. Only the spin unpolarized case is considered. Generalization to the spin-polarized case is trivial and can be implemented by increasing the number of input neurons corresponding to the spin components appropriately.

The NN-E input parameters are electron density *n* and the square of the electron density gradient $\sigma =\langle \nabla n, \nabla n \rangle$. We use base-10 logarithmic transformation for preprocessing input features of NN-E. The input features for the NN-V are $\varepsilon _{xc}$, *n*, $\sigma$, $\gamma = \langle \nabla \sigma , \nabla n \rangle$ and Laplacian of the electron density $\Delta n$. All NN-V features except $\varepsilon _{xc}$ are standardized, i.e. converted to zero mean and unit variance (see Fig. 1). The choice of features for NN-V is due to the functional connection between $\varepsilon _{xc}$ and $V_{xc}$ in generalized gradient approximation (GGA), which contains the electron density, its gradients in various combinations and the Laplacian. In a real space grid the connection between potential and XC energy density looks as following^33^:

1 $$\begin{aligned} v_{\mathrm {xc}}^{\mathrm {GGA}}=& \varepsilon _{\mathrm {xc}}^{\mathrm {GGA}}+n \frac{\partial \varepsilon _{\mathrm {xc}}^{\mathrm {GGA}}}{\partial n}-g \frac{\partial \varepsilon _{\mathrm {xc}}^{\mathrm {GGA}}}{\partial g}-n g \frac{\partial ^{2} \varepsilon _{\mathrm {xc}}^{\mathrm {GGA}}}{\partial g \partial n}\nonumber \\& \quad -\frac{n}{g} \frac{\partial \varepsilon _{\mathrm {xc}}^{\mathrm {GGA}}}{\partial g} \nabla ^{2} n+\frac{n}{g^{2}} \frac{\partial \varepsilon _{\mathrm {xc}}^{\mathrm {GGA}}}{\partial g} \vec{g} \nabla g -\frac{n}{g} \frac{\partial ^{2} \varepsilon _{\mathrm {xc}}^{\mathrm {GGA}}}{\partial g^{2}} \vec{g} \nabla g, \end{aligned}$$

where $g(\mathbf {r}) \equiv |\varvec{\nabla } n(\mathbf {r})|$ and $\vec {g} \equiv \nabla n(\mathbf {r})$. In a broad sense, the choice of features is arbitrary and the only criterion for its success is the increase in the accuracy of NN.

At the first stage of training, only the NN-V is trained. In this case, the energy that is supplied to the input to the NN-V is obtained using the libXC^34^ package. The first stage aims to teach mapping between the $\varepsilon _{xc}$ and the corresponding potential, and the $\varepsilon _{xc}$ known in advance is used. In this case the following loss function is applied:

2 $$\begin{aligned} Loss = \frac{1}{N}\sum \limits _{i=1}^N(V_{xc}^{PBE}[i] - V_{xc}^{predicted}[i])^2. \end{aligned}$$

At the second stage, the NN-V weights are frozen, and only NN-E part is trained, but loss depends on the output of NN-V ($V_{xc}$). The second stage aims to train the neural network to map electron density, $\sigma$ and $V_{xc}$. Simultaneously, the frozen NN-V provides the correct connection between $\varepsilon _{xc}$ and $V_{xc}$. At this stage we additionally include boundary condition in the loss function:

3 $$\begin{aligned}Loss & = \frac{1}{N}\sum \limits _{i=1}^N[(V_{xc}^{PBE}[i] - V_{xc}^{predicted}[i])^2 \nonumber \\& \quad + (\varepsilon _{xc}^{predicted}(0,\sigma )[i] - 0)^2 \nonumber \\& \quad + (\varepsilon _{xc}^{predicted}(n, 0)[i] - \varepsilon _{xc}^{LDA}(n)[i])^2]. \end{aligned}$$

The first boundary condition is based on the fact that $\varepsilon _{xc}$ tends to zero at zero density with any input $\sigma$. The second boundary condition follows from the fact that with zero $\sigma$ and any density $\varepsilon _{xc}$ leads to the corresponding energy of the local density approximation.

To obtain the training data, we carry out DFT calculations^1,2^ of silicon, benzene, and ammonia. We perform the calculations in real space using the Octopus code^29–31^. The XC functionals used include the exchange and correlation parts from PBE^10,28^. For all atomic species pseudo-potentials SG15 are used^35^. The total number of the calculations is 10 for each chemical substance. Atomic configurations in these calculations are differed by applied strain $\pm 5 \%$. Grid spacing in the range from zero to 10 is used. This corresponds to $64 \times 64 \times 64$ mesh for silicon and benzene, and $65 \times 65 \times 65$ mesh for ammonia. The mesh size for ammonia is a little bit larger due to technical issues related to non-periodic boundary conditions. For avoiding gradient inconsistency in the boundaries we use cropping. We remove data points lying at a distance of 4 (due to numerical scheme of differentiation) or less from the boundaries of the parallelepiped. Finally, the dataset size was approximately 5.2 million samples.

**Figure 3:**
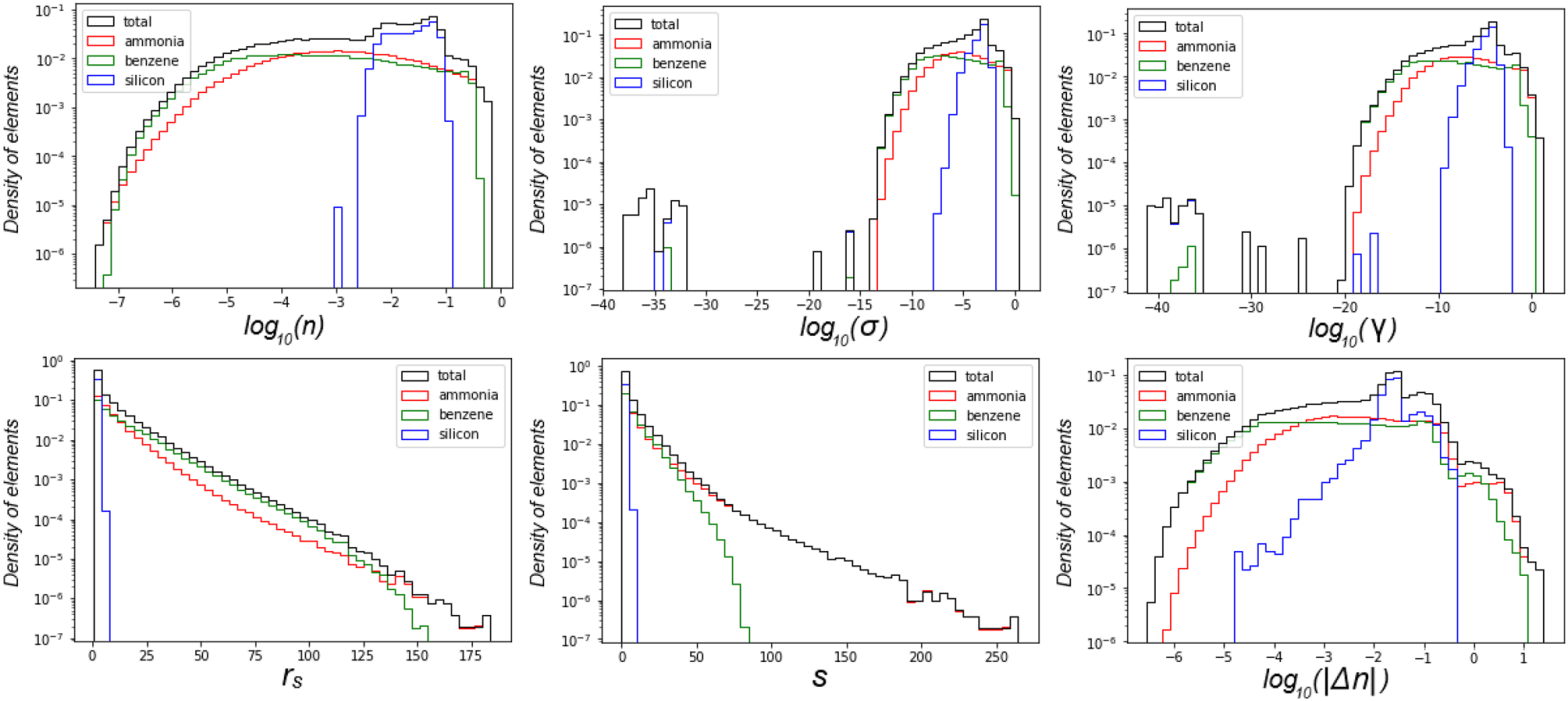
Distributions of input features. Each curve is normalized to to the total number of elements in the corresponding dataset.The distributions localized around the values that are typical for the systems used in training dataset: ammonia, benzene, crystalline silicon. One can see that crystalline silicon has relatively small electron density gradients and no region of low electron density due to its periodic structure. If a neural network is given an input value significantly out of bounds of the distribution data, it potentially could output an inappropriate value.

The Pytorch framework^36^ is utilized for training the neural network. We use the Adam algorithm for training with a learning rate descending from 0.001 by 25 percent every 20 epoch, and mean square error (MSE) loss. The batch size selected for training is 5000. The neural architecture used for the NN-E is a fully connected network with two input neurons, one linear output neuron, and four exponential linear units (ELU)^37^ hidden layers with 100 neurons. NN-V has the same architecture except that the number of input neurons increased to 5. To implement boundary conditions (3) we also included two additional batches in each training step. The first one consists of zero electron density and non-zero gradients with corresponding zero $\varepsilon _{xc}$. The second one contains non-zero electron density and zero gradients with corresponding $\varepsilon _{xc}^{LDA}$. Thus, on the one hand, we have included the boundary conditions directly in the loss function; on the other hand, we have reinforced the boundary conditions directly with data.

## Results

### Training of neural network

The training of NN was performed on benzene, silicon and ammonia. For each substance ten calculation was performed. In each calculation the system was stretched or compressed in range of $\pm {5 \%}$ of lattice constant to obtain electron densities in a wider range.

Training curves for NN-V and NN-E are presented in Fig. 2. The loss reaches a plateau after 300k batches for NN-V and 200k batches for NN-E correspondingly. Each batch consisted of 32 sample. The final loss achieved values of about $10^{-6}$ $\mathrm {Ha}^{2} \times a_{0}^{6}$ in both cases.

The distributions of input features for training dataset are presented in Fig. 3. Individual distributions of substances included in the training dataset are normalized to the total number of elements corresponding to the given substance. In additional to features used distribution by the Wigner–Seitz radius $r_{s}=\left( 3/(4 \pi n)\right) ^{1/3}$ and the reduced density gradient $\mathrm {s}=|\nabla \mathrm {n}| / 2 \mathrm {k}_{\mathrm {F}} \mathrm {n}=|\nabla n| / (2 \root 3 \of {3} \pi ^{2/3}n^{4/3})$ are presented. All distribution is not uniform due to real systems used in training procedure. The distributions localized around the values that are typical for the systems under consideration. Analysis of such distributions is important to determine the limits of NN XC functional applicability, i.e. to detect cases where NN XC functional will certainly fail due to absence of such data in the training dataset. This is especially important for case of heavy atoms in which core electron density could have large derivatives.

**Table 1:** MSE and MAE results for $\mathrm {\varepsilon _{xc}}$ and $\mathrm {V_{xc}}$ on a training dataset. The units of MSE are $\mathrm {Ha}^{2} \times a_{0}^{6}$ , the units of MAE are $\mathrm {Ha} \times a_{0}^{3}$.

	MSE ($\varepsilon _{xc}$)	MAE ($\varepsilon _{xc}$)	MSE ($V_{xc}$)	MAE ($V_{xc}$)
Benzene	$4\times 10^{-7}$	$2\times 10^{-4}$	$1\times 10^{-6}$	$6\times 10^{-4}$
Silicon	$2\times 10^{-5}$	$1 \times 10^{-3}$	$1\times 10^{-6}$	$4\times 10^{-4}$
Ammonia	$2\times 10^{-6}$	$4\times 10^{-4}$	$3\times 10^{-6}$	$1\times 10^{-3}$

**Figure 4:**
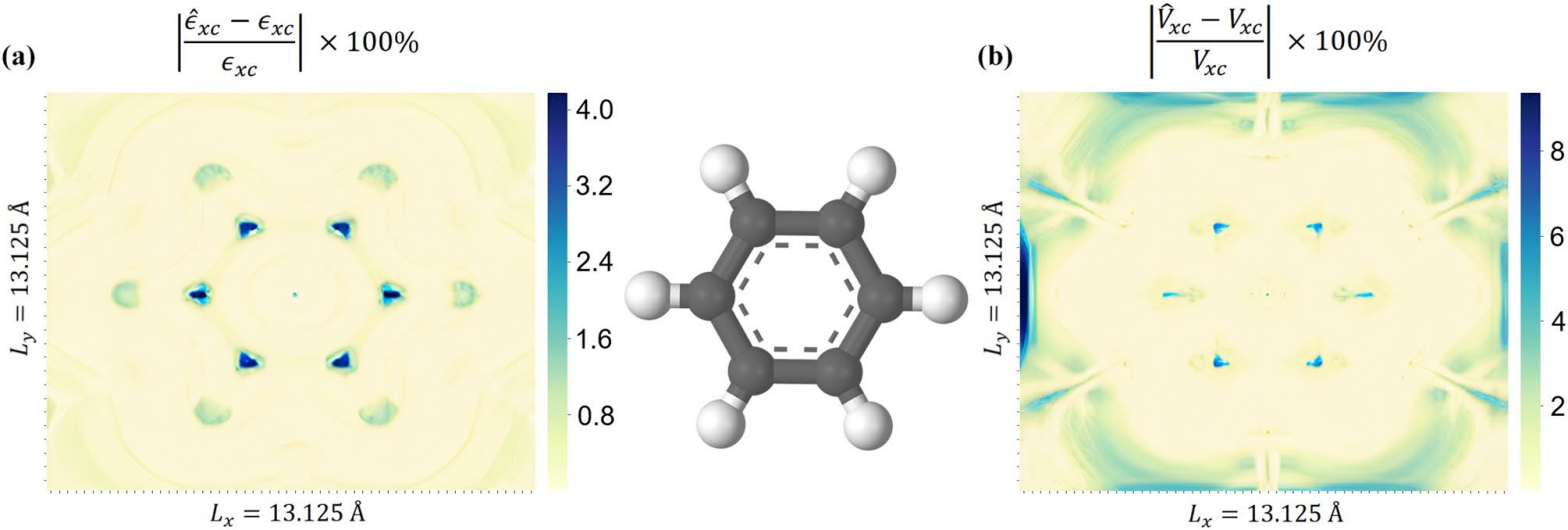
Spacial distribution of relative error for benzene: (**a**) spacial distribution of relative error for $\varepsilon _{xc}$, (**b**) spacial distribution of relative error for $V_{xc}$. The relative local errors have highest values in the region of the nuclei and at the boundary.

**Table 2:** Results of testing NN XC functional on a subset of IP13/03 dataset. $\mathrm {E_{total}}$ and $\mathrm {E_{xc}}$ denote total energy and exchange-correlation energy correspondingly obtained after convergence.

Substance	Error ($E_{total}$), $\%$	$Error (E_{xc}$), $\%$
C	0.052	0.287
S	0.015	0.102
SH	0.021	0.007
Cl	0.010	0.035
OH	0.554	1.036
$\mathrm {Cl_2}$	0.001	0.035
O	1.556	4.029
P	0.008	0.071
$\mathrm {O_2}$	0.646	2.482
PH	0.014	0.050
$\mathrm {PH_2}$	0.011	0.023
$\mathrm {S_2}$	0.023	0.040
Si	0.003	0.253

We use absolute mean square error and (MSE) and mean average error (MAE) as metrics to evaluate learning outcomes:

4 $$\begin{aligned} MSE(x)&= \frac{1}{N}\sum _{n=1}^{N} \left( \hat{x}_{n} - x_{n} \right) ^{2}, \end{aligned}$$

5 $$\begin{aligned} MAE(x)&= \frac{1}{N}\sum _{n=1}^{N} \left| \hat{x}_{n} - x_{n} \right| , \end{aligned}$$

where $x_n$ is the true value of target variable, $\hat{x}_n$ is the predicted value of target variable, *N* is the total number of targets. The resulting MSE and MAE on training dataset are presented in the Table 1. Minimum $\mathrm {MAE(}\varepsilon _{xc}\mathrm {)}$ and $\mathrm {MSE(}\varepsilon _{xc}\mathrm {)}$ are obtained on benzene, maximums are achieved on silicon. MSE error on boundary conditions is on the order of $10^{-10}$, which is considerably less than the total MSE error on the train dataset.

You can see in Fig. 3 that the distributions of silicon are significantly different from the overall distributions of the training dataset. One can see that crystalline silicon has relatively small electron density gradients and no region of low electron density due to its periodic structure. Therefore we attribute the maximum error of $\varepsilon _{xc}$ in silicon to the fact that its characteristic values of electron density and its derivatives effectively have a smaller fraction in the training dataset compared to benzene and ammonia.

We also separately analyzed the spatial distribution of the error using benzene as an example. Figure 4 shows the distribution of the relative local error $|({\hat{x} - x})/{x}| \times 100 \%$ in the benzene plane for both $\varepsilon _{xc}$ and $V_{xc}$. The spatial distribution of the error clearly shows that it has the highest values in the region of the nuclei and at the boundary. Again, we associate the high local error in these places with a few examples of this type of data in its training set. If there were more extreme examples with high and low density in the training dataset, then the errors in the nuclei region and at the border should have dropped.

**Figure 5:**
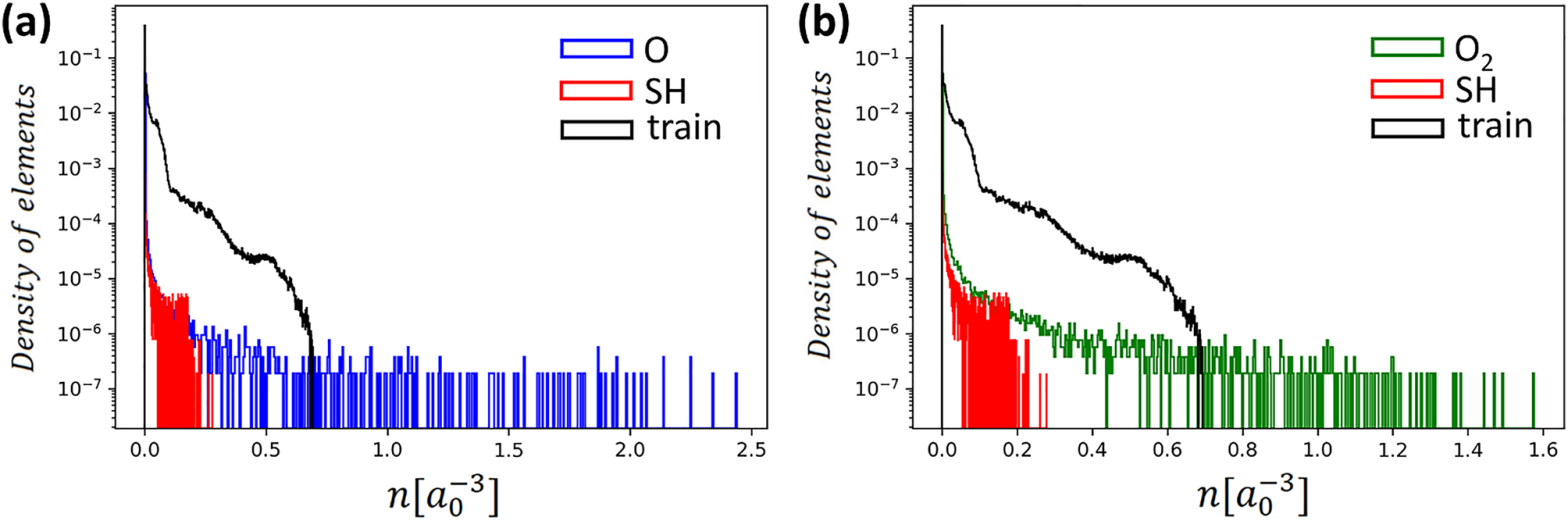
Comparison of distribution for non self-consistent electron density of atomic oxygen (**a**), molecular oxygen (**b**), hydrogen sulfide and self-consistent electron density from training ($a_{0}$ denotes the Bohr radius). One can see that atomic and molecular oxygen have a significant fraction of samples out of the train distribution. The step of the self-consistent cycle with maximum electron density was chosen for all substances. This example emphasizes the importance of including a wide range of electron density and derivatives in the training dataset because extreme values could occur during the self-consistent cycle.

**Table 3:** Results of testing NN XC functional on Alkisomer11 dataset. $\mathrm {IE}$ denotes isomerization energy.

Isomerization reaction	IE (PBE)	IE (NN)	Abs error, kcal/mol
Butane $\rightarrow$ isobutane	− 0.996	− 1.090	0.094
Pentane $\rightarrow$ isopentane	− 0.788	− 0.491	0.297
Pentane $\rightarrow$ neopentane	− 2.315	− 1.843	0.472
Hexane $\rightarrow$ isohexane	− 2.563	− 2.071	0.492
Hexane $\rightarrow$ neohexane	− 2.516	− 1.924	0.592
Hexane $\rightarrow$ diisopropyl	− 1.626	− 0.896	0.730
Hexane $\rightarrow$ 3methylpentane	− 2.062	− 1.409	0.653
Heptane $\rightarrow$ isoheptane	− 3.218	− 2.916	0.302
Heptane $\rightarrow$ neoheptane	− 2.807	− 2.746	0.061
Octane $\rightarrow$ hexamethylethane	5.224	6.326	1.102
Octane $\rightarrow$ isooctane	0.725	1.292	0.567

### Testing of neural network

We incorporate the developed NN XC functional into Octopus code and perform self-consistent cycle calculations. All calculations are converged, and relative total and XC energies errors are calculated. As a reference, we take PBE functional that was used for the training of NN. Results are presented in the Table 2. One can see that the highest error is achieved for atomic and molecular oxygen.

**Figure 6:**
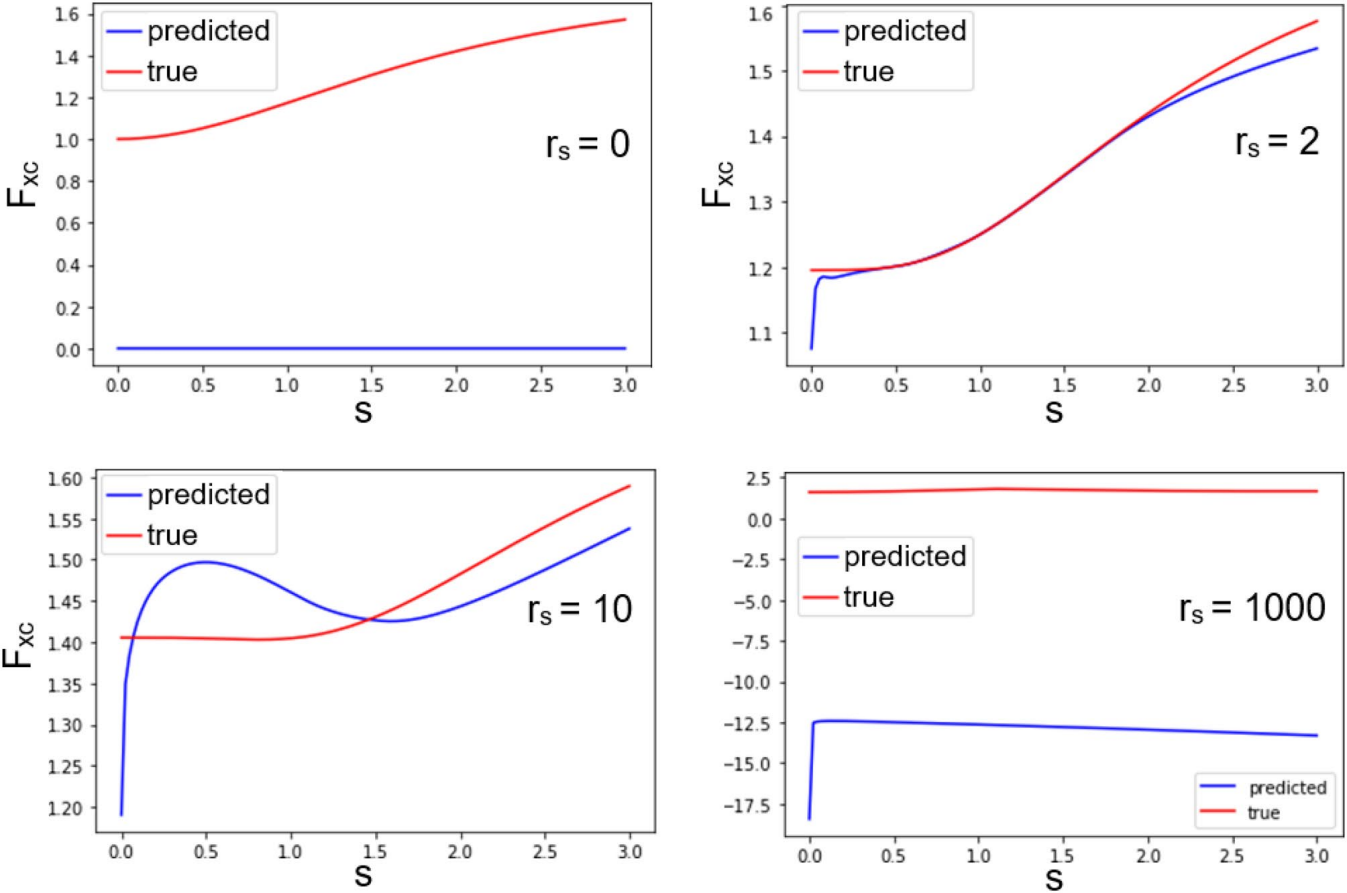
Enhancement factor $F_{xc}$ at fixed values of $r_{s}$: 0, 2, 10, 1000. *s* is a reduced density gradient. There is a good agreement between the enhancement factors at $r_s$ = 2 densities, which is close to the most frequent densities in the training set. Also one can see that NN XC functional is bad in guessing asymptotic in low ($r_s$ = 1000) and high ($r_s$ = 0) limits of electron density.

The reasons for the error on oxygen were analyzed. It turned out that the main reason for such a discrepancy on some substances, including oxygen, is the intermediate densities that occurred in the process of a self-consistent cycle that the neural network did not see during the training process. The problem is that such densities can be arbitrarily large. The distributions of maximum electron densities in some cases are presented in Fig. 5 together with the training distribution. Analytical exchange-correlation functional contains asymptotics at infinity, which makes it possible to process such cases correctly. However, only boundary conditions at zero were used during our training cycle, but asymptotics at infinity were not used. This critical fact describes obtained discrepancy and gives hope for the further development and improvement of neural network exchange-correlation functionals by including such asymptotics^38,39^.

To show the performance of developed NN XC on a chemically meaningful quantity we included results for Alkisomer11^11^ dataset (see Table 3), for which we made PBE vs NN XC comparison of isomerization energies. Our model demonstrates results comparable to PBE, mean absolute error (MAE) for PBE is 1.6 kcal/mol and MAE for NN XC is 1.7 kcal/mol, which is a quite promising result. As it turned out as a result of the analysis, large errors on octane isomers even for PBE are explained by the fact that the corresponding geometries published in the article^11^ are not completely relaxed.

To verify the importance of nonequilibrium densities for convergence of the SCF cycle we expanded the training dataset to include higher densities typical for intermediate densities of oxygen atom (O), which are obtained in self-consistent cycles (see Fig. 5). We stopped SCF iteration at 2, 4, 6, 8, 10, 12, 14, 16, 18, and 20 iterations and added 10 corresponding SCF results to the training set. We used spacing 0.273 $a_{0}$ and $64 \times 64 \times 64$ mesh. The resulting neural network performed quite better for O$_2$, the relative error of total energy drops from 0.646 to 0.023% and the relative error of XC energy drops from 2.5 to 0.17%. Thus, we also have shown that the inclusion of additional data, such as nonequilibrium densities (intermediate densities in the SCF cycle), makes it possible to increase the accuracy of the XC functional.

Enhancement factor $F_{xc}(r_s, s) = \varepsilon _{xc} / \varepsilon _{x}^{LDA}$ obtained from NN-E prediction was also analyzed, where *s* is a reduced density gradient $s=|\nabla \mathrm {n}| / 2 \mathrm {k}_{\mathrm {F}} \mathrm {n}=\frac{\left( \frac{1}{n}\right) ^{4 / 3}|\nabla n|}{2 \root 3 \of {3} \pi ^{2 / 3}}$. It compared with corresponding $F_{xc}$ obtained from prediction of analytical exchange-correlation functional (PBE) by libxc (see Fig. 6). There is a high similarity between the enhancement factors at densities that closely match the most frequent densities in the training set ($r_s$ = 2). We observe slightly less similarity at $r_s = 10$. This can be explained by the decreased number of elements with such a density in the training set. A significant discrepancy is observed in the limiting cases of high and low densities. In the case of high density (small $r_s$), this is explained by the absence of asymptotic at infinity. Despite the presence of asymptotic at zero densities in the process of training a neural network, the obtained result shows that this asymptotic may not be enough for obtaining a good enhancement factor.

**Figure 7:**
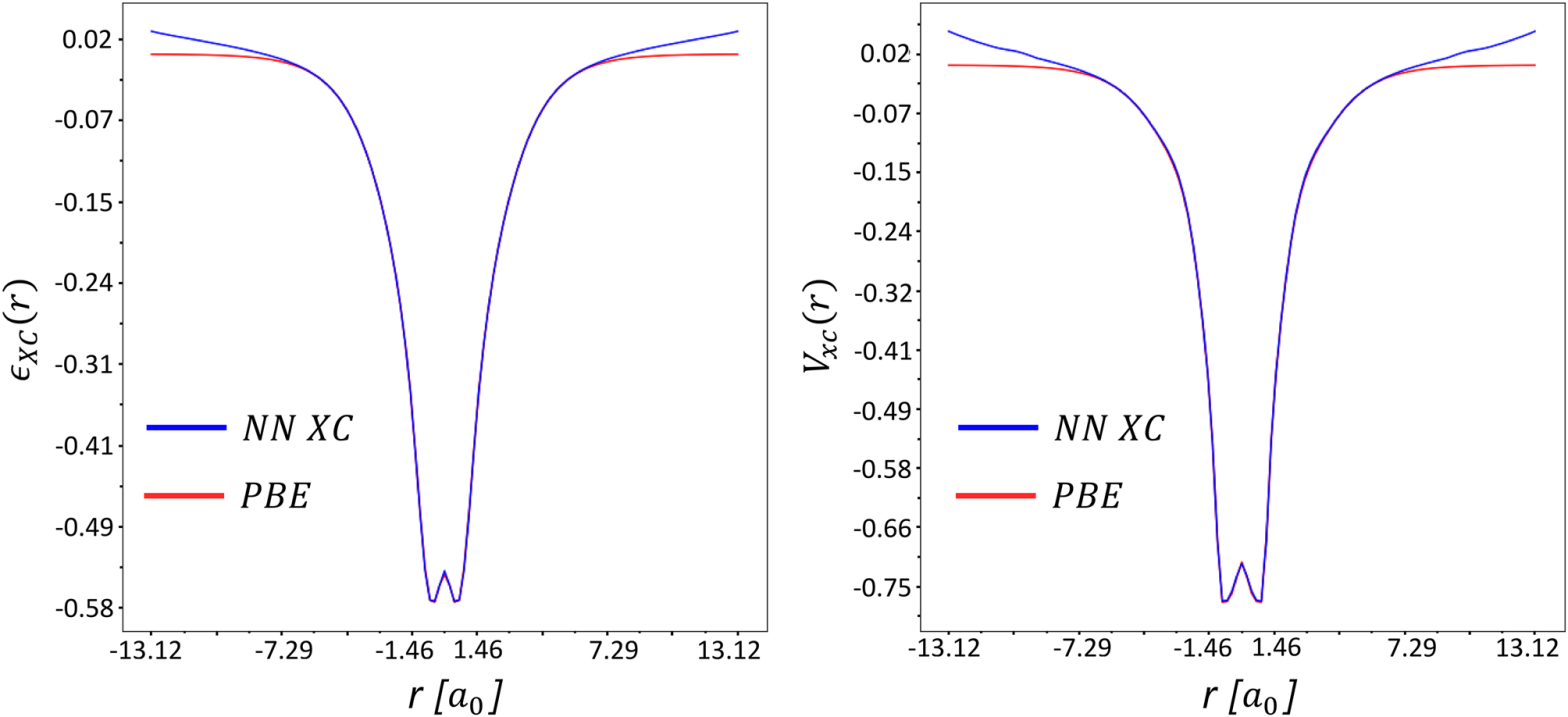
Slices of $\varepsilon _{xc}$ and $V_{xc}$ along the line connecting the atoms of a hydrogen molecule. Blue lines represent values predicted by our NN, red lines—values obtained by Octopus (where $V_{xc}$ is correct functional derivative).

**Table 4:** Comparing NN and PBE energies of rotated SH molecule. All energies in Hartree.

Rotation (deg.)	Total energy (PBE)	Total energy (NN)	XC energy (PBE)	XC energy (NN)
0	− 10.69546	− 10.69320	− 2.48893	− 2.48901
11.25	− 10.69546	− 10.69321	− 2.48891	− 2.48911
22.5	− 10.69542	− 10.69314	− 2.48890	− 2.48892
45	− 10.69544	− 10.69314	− 2.48892	− 2.48905
90	− 10.69546	− 10.69321	− 2.48893	− 2.48901

Even though our model correctly reproduces the energies, the true exchange-correlation functional must satisfy many important physical constraints. Therefore, it is very crucial to discuss how such constraints can be added to the model and learning process in our future work. Firstly, it is important to mention that constraints use total exchange or correlation energies, so correct implementation of constraints can be effectively introduced only while training the model on total energies (much better, reaction energies). For example, an important property of such a functional is the density scaling property. In the article^40^, it was shown that this property can be effectively taken into account using contrastive representation learning. Concerning our model, this means that the densities and all values obtained from it should be fed into NN-E and NN-V not in a “pure” form, but transformed using an encoder trained in an unsupervised way. As stated in the article mentioned above the NT-xent loss is a good choice. This loss causes the projected representations of unscaled and scaled densities of the same molecule to get close and the representations of different molecules to be far from each other. With another module predicting scaling factors this approach can help to incorporate uniform density scaling constraint. In the case of our work, we did not actively investigate the imposition of any constraints, this is the topic of ongoing research, but we checked that the presented functional does not lose accuracy when the SH molecule was rotated (see Table  4). As a result of additional calculations, it turned out that our model demonstrates excellent rotational equivariance with a total energy difference significantly smaller than 1 kcal/mol.

XC energy density and the XC potential are related with a functional derivative (see for example Eq. 1) Therefore, we compared the exchange-correlation potential predicted by the NN-V and the corresponding potential calculated using the functional derivative. Indirectly, the fact that the analytical relationship between $\varepsilon _{xc}$ and $V_{xc}$ is preserved can be shown by the fact that the errors both on energy and on potential are quite small at the same time (see Table 1). If the neural network did not correctly reproduce this connection, then, accordingly, the error on the potential would be quite large. We aslo demonstrated these results clearly on the hydrogen molecule and built distributions of $\varepsilon _{xc}$ and $V_{xc}$ along the line connecting the hydrogen atoms (see Fig. 7).

## Conclusions

The second NN (such as NN-V) that directly predicts the XC potential is avoided in recent publications^26,41^, by imposing automatic differentiation of the NN. Automatic differentiation is a remarkable property of neural networks, which is actively used for developing neural network exchange-correlation potentials. However, it has some disadvantages: if the neural network is deep enough then automatic differentiation makes much noise, especially if we talk about higher-order derivatives (most of the implementations in literature use DFT codes based on atomic orbitals, where only first-order derivatives are needed); for simple features, it is pretty straightforward to write an analytical form of the relationship between the potential and the XC energy. However, if we take complex and non-standard features (semi-local,global ones), it can be practically impossible to connect the potential and the exchange-correlation energy. This is where the advantage of the second neural network becomes important—it provides an alternative path for an approximation that does not involve taking partial derivatives. This allows using more complex features in the machine learning pipeline.

Low error on the validation dataset indicates that the developed approach to the architecture of the XC functional interpolates well the existing XC functionals and has the high generalizing ability. Furthermore, in the framework of the proposed architecture, one can train NN-E and NN-V separately, making it flexible to use. The basic strategy to create working functional in the framework of the proposed architecture would be initially to train the NN-V part on a specific type of existing XC functional such as LDA, GGA, or meta-GGA, using various types of input features. Then the NN-E part of the neural network is trained on the data obtained by accurate post-Hartree–Fock or quantum Monte Carlo methods.

In this work, we did not take into account that the resulting functional may violate the rules of critical physical conditions^41,42^. However, all these conditions can be introduced by modifying the training loop or loss function and, therefore, can be explicitly taken into account. This is a matter for further research.

The main advantage of the NN approach in comparison with other interpolation techniques for XC functionals is its flexibility to incorporate exchange-correlation data from different sources, such as post-Hartree–Fock and quantum Monte Carlo. It is possible that application of the NN to interpolate high-level XC quantum data could eliminate many heuristics used in the traditional construction of XC functionals.

## Data Availability

Optimized NN weights and code to use it in calculations are available at https://github.com/AlexanderFreeman/octopus-nn-2.
